# Total Hip Arthroplasty in a Patient With Contralateral Hemipelvectomy: A Case Report With a One-Year Follow-Up and Literature Review

**DOI:** 10.7759/cureus.97620

**Published:** 2025-11-23

**Authors:** Eteesha Rao, P Nithin Unnikrishnan

**Affiliations:** 1 Surgery, Newcastle University, Newcastle Upon Tyne, GBR; 2 Orthopaedic Surgery, Robert Jones and Agnes Hunt Orthopaedic Hospital NHS Foundation Trust, Oswestry, GBR

**Keywords:** hemipelvectomy, hindquarter amputation, surgical positioning, total hip arthroplasty, total hip replacement

## Abstract

Total hip arthroplasty (THA) is a standard procedure to alleviate pain and improve joint function in patients with advanced degenerative hip osteoarthritis. Hemipelvectomy, or hindquarter amputation, is a complex, technically demanding procedure with a challenging prosthetic rehabilitation journey. It is often indicated for malignancies or traumatic injury. Hemipelvectomy often leads to a lack of structural stability and altered weight distribution, requiring customized approaches to ensure stability and functional restoration. Thus, THA in patients who have undergone contralateral hemipelvectomy remains a rare procedure, which presents unique surgical and biomechanical challenges, questioning its feasibility in improving the patient’s quality of life. This report presents the surgical management and one-year outcomes of a 60-year-old male with a severely osteoarthritic left hip who underwent right-sided hemipelvectomy due to trauma at the age of 18 years, 42 years ago. We then compared our clinical data with other reports in the medical literature, comparing the management approaches with functional outcomes. A literature review was conducted using electronic databases, including PubMed and Google Scholar. Comparisons were drawn with our case to the management approaches with immediate and long-term functional outcomes. A review of the past two decades of literature on THA in similar patients revealed a series of successful procedures with improved long-term mobility. This has been due to significant advancements in surgical positioning, implant technology, and postoperative care, all aimed at optimizing outcomes in this high-risk population. Our findings highlight the feasibility and positive impact of THA in hemipelvectomy patients through a carefully tailored approach with multidisciplinary coordination. While positive outcomes are increasingly achievable, continued research into long-term solutions and adaptive rehabilitation protocols is essential.

## Introduction

Total hip arthroplasty (THA) is a standard procedure to alleviate pain and improve joint function in patients with end-stage degenerative hip osteoarthritis [1]. It can also be performed for patients with other hip pathologies, such as hip osteonecrosis, developmental hip dysplasia, and inflammatory arthritis [2]. However, THA in patients who have undergone contralateral hemipelvectomy represents a complex intersection of biomechanical alteration and surgical challenge.

Hemipelvectomy, also referred to as hindquarter amputation, is often required for malignancies or traumatic injury [3,4]. It is a complex, technically demanding procedure with a challenging prosthetic rehabilitation journey. Hemipelvectomy often leads to a lack of structural stability and altered weight distribution [5]. These anatomical shifts complicate typical THA approaches and increase the risk of complications, including implant loosening, dislocation, and infection [4].

This case report and literature review provide a comprehensive look at both a recent complex THA case and the cumulative knowledge in this area over the last 20 years, aiming to elucidate best practices and discuss the ongoing evolution of surgical techniques and patient outcomes.

## Case presentation

A 60-year-old male presented with a three-month history of severe left hip pain that had progressively restricted his mobility. He had sustained a traumatic right hindquarter amputation 42 years earlier, at the age of 18. The patient’s condition had been stable, but increasing left hip pain rendered him nearly housebound, forcing him to quit his labor-intensive profession. He was limited to short distances of 50-60 feet with the aid of two crutches. His pain was controlled using analgesics such as paracetamol and buprenorphine patches only due to a history of gastrointestinal bleeding with non-steroidal anti-inflammatory drugs. Additionally, the patient exhibited heavy smoking and alcohol use as coping mechanisms, exacerbating his health risks and complicating his clinical profile.

Differential diagnoses, investigations, and treatment

**Figure 1:**
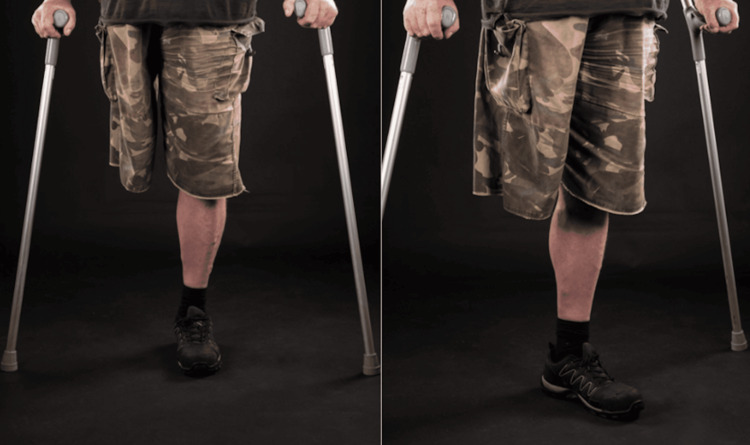
Preoperative mobility: standing upright with supporting crutches.

Preoperatively, on examination, the patient appeared to be well-built and mobilizing with the aid of two crutches (Figure 1). He had a well-healed right hindquarter amputation on inspection. On palpation, the patient had left-sided widespread tenderness across the hip, predominantly in the groin, trochanteric area, and buttocks. His range of motion at the left hip joint was difficult to assess due to severe pain, but it was noted that the pelvis would move even on slight abduction or adduction. Log rolling was painful, and the patient had a flexion deformity of up to 50 degrees. Internal and external rotation were restricted and painful for the patient.

**Figure 2:**
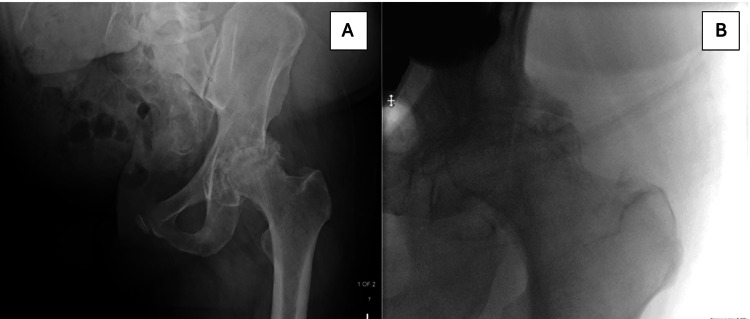
Preoperative X-ray of the left hip. A: Lateral view of the left hip joint showing osteoarthritic changes. B: Anterior view of the left hip joint showing osteoarthritic changes.

X-ray imaging (Figure 2) showed advanced osteoarthritis with bone-on-bone contact in the left hip, confirming the need for surgical intervention. However, given his history of contralateral hemipelvectomy, the case presented considerable challenges in terms of operative technique and appropriate surgical positioning.

Taking into account the complex clinical picture and the impact of the pain on the patient’s quality of life, surgery was considered and presented to the patient as a high-risk option with no assurance of a predictable outcome. The patient was informed of his case-specific increased risks associated with surgery, particularly infection; higher chances of dislocation due to having a hemipelvis; higher risks of nerve, vessel, or tendon damage; and higher risks of wound complications. The patient was motivated and willing to accept these risks in an attempt to regain his mobility. Thus, he consented to undergo complex primary total hip replacement. After extensive discussion, the patient agreed to the proposed complex left hip replacement surgery.

**Figure 3:**
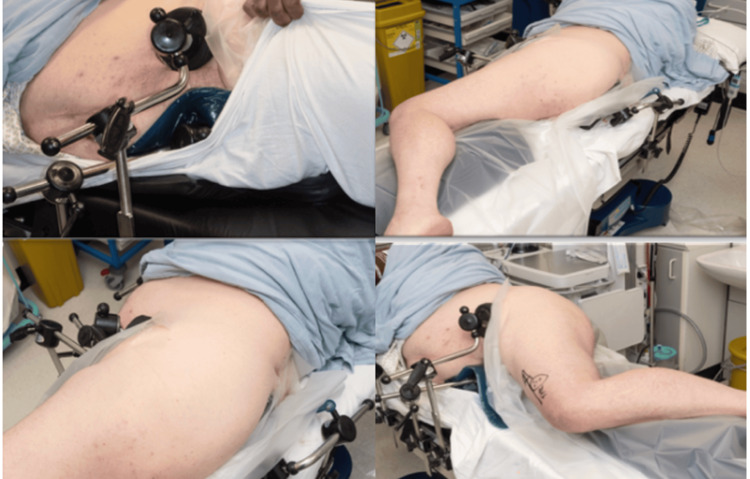
Intraoperative patient positioning for left total hip replacement. The marking on the patient’s thigh in the bottom right image is a surgical marking.

Given the intricacies involved, the case was presented at the departmental arthroplasty and multidisciplinary meeting, including orthopedic surgeons, rheumatologists, radiologists, and anesthetists. In an effort to minimize intraoperative complications, the team planned a trial positioning session in the operating theater weeks before surgery, under X-ray control. The patient provided written informed consent for this. The aim was to find the optimal position for the approach, allowing the best access and setup for the left total hip replacement. General anesthesia was used in this setting to ensure equipment availability of positioning supports and to allow for time and flexibility if new material were to be sourced from elsewhere (Figure 3).

In addition to this, the patient was advised to make substantial lifestyle adjustments, including smoking and alcohol cessation, to reduce the chances of infection and improve bone quality in the lead up to the operation. After multiple preoperative evaluations, the patient was deemed ready for surgery, having demonstrated adherence to the prescribed lifestyle changes.

The patient was admitted for an elective left complex primary hip replacement on the morning of the operation. The procedure was performed under spinal anesthesia, with the patient being given tranexamic acid and intraoperative antibiotics as per protocol.

The patient was laterally positioned with a sandbag placed under the right hemipelvis and supports as trialed in the theaters previously. Standard procedures were followed before commencing the operation, with double chlorhexidine preparation and exclusion drapes being used.

A posterior approach was used with care taken to protect the sciatic nerve. A capsular stitch was then used to establish length and offset. After this, a neck osteotomy was performed, and the head was extracted. The severity of the degenerative changes in this osteoarthritic left hip joint could be clearly visualized. The acetabulum was then sequentially reamed to 57 mm. The 57 mm bi-mentum cup had a good hold on trialing and continued to insert this aligned to the transverse acetabular ligament and with 40 degrees inclination. Osteophytes were removed.

The femoral canal was then opened and sequentially rasped to size 12. The surgeon trialed for a good length, offset, and stability with a high offset until they decided on a 28 + 8.5 dual mobility 28/57 liner. A Corail 12 high-offset collared stem and a 28 + 8.5 ceramic head 28/57 PE dual mobility liner were impacted and inserted. A washout was performed, and a local anesthetic was injected into the area. The incision was closed in layers with Vicryl through the drill holes, PDS, and quill. The skin was closed using clips and covered with an Aquacel dressing.

Postoperative course and follow-up

**Figure 4:**
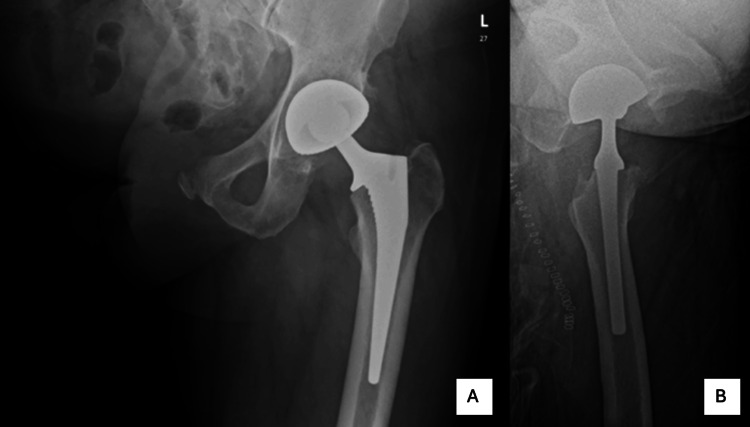
Postoperative X-ray of the left hip. A: Lateral X-ray of the left hip after total hip replacement. B: Anterior X-ray of the left hip after total hip replacement.

The patient was kept admitted on the wards for a further two days to allow for adequate observation and a gentler rehabilitation approach. He began to mobilize from day one after the complex left total hip replacement and was discharged with no concerns. The patient was advised to sleep on their back for 12 weeks and have the wound checked and clips removed at two weeks postoperatively (Figure 4).

The patient was discharged with instructions to sleep supine for 12 weeks. His postoperative course was uneventful, and at six weeks, he reported significantly improved mobility and pain relief. At one year, the patient remained highly satisfied, reporting restored quality of life and a return to independence.

## Discussion

A literature review was conducted to review the previous approaches for THA and their clinical outcomes in relation to patients with an osteoarthritic hip joint on a background of contralateral hemipelvectomy. We compared the findings with our case to assess the best management approach for this patient group.

Methodology

To achieve our aim, a literature search was conducted using electronic databases, including PubMed and Google Scholar. Key terms (such as hemipelvectomy, hindquarter amputation, total hip arthroplasty, total hip replacement) relevant to THA and their clinical outcomes in relation to patients with an osteoarthritic hip joint on a background of contralateral hemipelvectomy. Articles not presenting data for patients with total hip replacement with a background of contralateral hemipelvectomy/hindquarter amputation (due to any cause) were excluded. Only articles meeting the criteria of total hip replacement with a background of contralateral hemipelvectomy/hindquarter amputation were included. Case reports from all years (2006-2023) were included. Of the case reports obtained, four peer-reviewed primary sources, spanning 17 years of surgical practice in this field, met our inclusion criteria and are presented with comparisons to our case (Table 1).

**Table 1:** Case reports on total hip arthroplasty with a background of contralateral hemipelvectomy. *: Focuses on rehabilitation rather than direct surgical management.

Study	Patient’s age (sex)	Time since hemipelvectomy	Hemipelvectomy Indication	THA positioning and approach	Outcomes (immediate)	Follow-up period	Outcomes (reported at the end of the follow-up period)
Bong et al. (2006) [4]	62years (male)	39 years	Recurrent fibrosarcoma	Lateral decubitus positioning secured with pillows. A bump was placed under the pelvis, and pillows were placed under the leg to help prevent adduction of the right hemipelvis and limb. Posterior surgical THA approach	Well-tolerated and uncomplicated operation and admission. Fully weight-bearing and mobilizing independently with axillary crutches (optimum for the patient). Discharged home on day four postoperatively	1y	The hip was stable in all ranges of motion. Postoperative radiograph found the acetabular component to be more vertical than desired
Sommerville et al. (2006) [6]	62years (female)	10 years	High-grade spindle cell sarcoma	Positioned on the right side with the spine parallel to the operating table and with the remaining hemipelvis vertical to this. Lateral position kept with the use of a vacuum beanbag molded to the patient’s shape. Lateral surgical THA approach	Mobilized from day five postoperatively with physio support, including a hydrotherapy pool. Mobilizing safely and discharged on day 20 postoperatively	4 years	At 3 months: fracture through the floor of the acetabulum, treated conservatively with rest in a wheelchair. Four-year review: the replaced hip caused no pain, and examination showed a full range of movement. X-rays showed that the fracture of the floor of the acetabulum had healed, but the cup had migrated slightly proximally. Tended to have an adducted leg, but there were no radiological or clinical features suggestive of subluxation
Goyal et al. (2009) [7]	52 years (male)	43 years	Trauma	Lateral decubitus position with the aid of a sandbag and multiple pillows secured under the leg to prevent marked adduction of the leg. Posterolateral surgical THA approach	Partially weight-bearing for the initial six months	5 years	No pain and walking unlimited distances with two crutches without a contralateral prosthesis. Radiographs showed a well-fixed implant, maintenance of graft, and no evidence of loosening/wear
Mazabel et al. (2023) [8]	42 years (male)	16 years	Trauma	Lateral decubitus with several pillows placed under the hemipelvis to avoid adduction. Posterolateral surgical THA approach	The patient was discharged 48 hours after surgery patient after meeting rehabilitation goals and achieving adequate pain control	6 years	No complications and adequate functionality with complete mobility of the operated hip joint

Each case underscores key elements in preoperative planning, implant choice, and rehabilitation tailored to these patients. Here, we review cases reported by Bong et al. (2006), Sommerville et al. (2006), Goyal et al. (2009), and Mazabel et al. (2023) [4,6-8].

Bong and Sommerville each documented THA in 62-year-old patients with a history of hemipelvectomy for oncological reasons, who developed contralateral hip osteoarthritis after 39 and 9 years, respectively. Both patients were positioned laterally, but Bong et al.’s team used an uncemented prosthesis with acetabular screws, while Sommerville et al.’s team opted for a cemented implant. These cases reflect a strategic approach to implant fixation based on bone quality and patient stability needs. At the one-year follow-up, both patients reported positive outcomes; however, Sommerville et al.’s patient experienced a fracture through the floor of the acetabulum, which consolidated without further issues, emphasizing the potential for bone-related complications in such cases [4,6,9].

Goyal et al. and Mazabel et al.’s cases documented a 52-year-old and 42-year-old patient, respectively, with a history of traumatic hemipelvectomy who developed hip osteoarthritis after 40 and 16 years, respectively. This case followed the lateral decubitus positioning, with supports to stabilize the hemipelvis and prevent adduction, combined with a posterolateral surgical approach. Using a posterolateral approach, Goyal addressed a significant acetabular defect with autograft and screw fixation before implanting a non-cemented prosthesis with an extended femoral stem. Their five-year follow-up indicated excellent functionality and stable outcomes, showing the benefits of grafting in managing complex acetabular defects [7]. Mazabel et al.’s patient was discharged 48 hours postoperatively after achieving pain control and rehabilitation goals. At the six-year follow-up, the patient reported full mobility in the hip joint without complications, further illustrating that with flexible, adaptive techniques, appropriate support, and stable implant placement, sustained functional outcomes are achievable in patients with extensive skeletal alterations [8].

The case literature indicates generally positive outcomes for THA in hemipelvectomy patients, but the procedure is not without risk. Complications commonly include implant loosening, dislocation, and infection, largely attributable to altered biomechanics and difficulties achieving stable fixation. The case we have presented here supports findings from the literature, as careful preoperative planning and individualized implant selection can significantly reduce such risks [9]. Long-term follow-up studies remain sparse, though available data show promising implant retention rates and sustained functional improvement when comprehensive postoperative care is implemented [4,7,8].

Discussion

THA in patients with a contralateral hemipelvectomy is rare, and documented cases highlight the unique anatomical and biomechanical complexities involved in these procedures.

A commonality among these cases is the use of lateral decubitus positioning, reinforced by the addition of pillows or specialized supports, to reduce adduction, optimize stability, and surgical access [10]. While Bong et al. and Sommerville et al. preferred a lateral approach to avoid risk of prosthetic dislocation, recent cases by Goyal et al. and Mazabel et al. opted for the posterolateral approach, which has gained favor due to its potential to reduce dislocation rates [11,12].

Implant fixation approaches varied based on the patient’s bone quality and structural needs [13]. In Bong et al.’s and Goyal et al.’s cases, non-cemented components with screw fixation were chosen for their potential to integrate with the bone and enhance long-term stability [4,6]. Sommerville et al.’s use of a cemented prosthesis addressed different stability needs, likely dictated by bone quality [7]. On the other hand, Mazabel et al.’s report reflects a continued trend toward non-cemented components in patients with strong cortical bone, which promote natural osseointegration and reduce the need for additional fixation materials [8,13]. In cases with significant bone loss, as with Goyal et al.’s case, the use of autografts to fill acetabular defects provided necessary structural support for implant stability [6,14]. Modular implants and extended femoral stems further reinforced these structures, showing the effectiveness of these modern, customizable components in accommodating compromised bone quality and extensive skeletal adaptations [15].

Rehabilitation outcomes across these cases were promising, with all patients achieving good pain relief and increased mobility. Each patient in the review used crutches for ambulation and reported substantial functional improvements [4,6-8]. Long-term follow-up is crucial to monitor implant performance and address any emerging complications.

## Conclusions

THA in patients with contralateral hemipelvectomy remains a challenging yet feasible procedure with significant potential for pain relief and functional improvement. This case report and literature review provides insights into best practices, emphasizing the importance of multidisciplinary coordination, innovative positioning strategies, and patient-specific implant choices to achieve excellent outcomes even in high-risk scenarios. While positive outcomes are increasingly achievable, continued research into long-term solutions and adaptive rehabilitation protocols is essential to further improve care for this unique patient population.
